# Changes in Growth and Feeding Characteristics during Early Ontogenesis in Threadsail Filefish, *Stephanolepis cirrhifer*

**DOI:** 10.3390/ani13213420

**Published:** 2023-11-04

**Authors:** Wengang Xu, Jun Zeng, Weiping Mei, Lianglong Jiang, Soichiro Manabe, Yanqin Wu, Liming Liu

**Affiliations:** 1 School of Ocean, Yantai University, Yantai 264005, China; xugang3041@163.com (W.X.); jianglianglong1997@163.com (L.J.); 2 Guangxi Academy of Sciences, Nanning 530007, China; junzeng@gxas.cn (J.Z.); mei@gxas.cn (W.M.); 3 Institute of Beibu Gulf Marine Industry, Fangchenggang 538000, China; 4 Graduate School of Fisheries and Environmental Sciences, Nagasaki University, Nagasaki 852-8521, Japan; 5 East China Sea Fisheries Research Institute, Chinese Academy of Fishery Sciences, Shanghai 200090, China; wuyanqing0961@163.com

**Keywords:** growth indices, point-of-no-return, feeding incidence, satiation, diel feeding rhythm, *Monacanthidae*

## Abstract

**Simple Summary:**

*Stephanolepis cirrhifer*, a commercially important marine fish, has been listed on the IUCN Red List of Threatened Species owing to a sharp decline in its wild-caught numbers. To increase the survival rate of juveniles after hatching, the changes in growth and feeding characteristics during early ontogenesis in this species must be understood. In this study, the growth indices at 24, 28, 30, 40, 45, and 50 days post hatching (dph) were significantly higher than those at earlier time points, suggesting that the juveniles possessed well-developed digestive organs and that a compound feed is suitable for rapid growth at these stages. The obligatory mixed-nutrition period was brief, at only 3–4 dph, and the larvae reached the point-of-no-return (PNR) at 1.5–2 dph. Furthermore, the growth indices of juveniles fed only under light conditions showed clear peaks and troughs. This is indicative of daytime feeding behaviour. These results serve as a reference to guide larval rearing and feeding programs that can improve the survival rates of *S. cirrhifer*.

**Abstract:**

Background: We investigated the growth and feeding characteristics of threadsail filefish, *Stephanolepis cirrhifer*, during early ontogenesis. Methods: The growth indices of hatchlings fed compound feed were measured from 0 to 50 days post hatching (dph). The absorption time of the yolk sac and oil globule, as well as the rate of first feeding were measured to characterise the early growth stage and determine the point-of-no-return (PNR). Feeding characteristics and rhythms were investigated under a light/dark cycle and under continuous light. Results: Growth indices increased significantly at 24, 28, 30, 40, 45, and 50 dph. The yolk sac and oil globules were completely absorbed before 4 dph, indicative of a short mixed-nutrition period at 3–4 dph. Under starvation conditions, the first feeding rate was highest (86%) at 0.5 dph and then decreased to 53.3% at 1.5 dph and 26.2% at 2 dph, suggesting that the PNR occurs at 1.5–2 dph. The feeding peak appeared at 15:00–18:00 and under light conditions, while the feeding trough appeared at 0:00–3:00. Conclusions: Compound feed supplied adequate nutrition for early growth and development. The peaks and troughs of feeding times were indicative of daytime feeding behaviour. These results provide guidance for successful rearing of filefish seedlings and juveniles.

## 1. Introduction

The threadsail filefish, *Stephanolepis cirrhifer*, is a commercially valuable marine species distributed throughout the East China Sea off the coasts of China, Japan, and Korea [1,2,3]. Due to overfishing and a high market demand, the wild-caught output of *S. cirrhifer* decreased by as much as 99% between 1990 and 2009 in South Korea [4]. Accordingly, the species was added to the IUCN Red List of Threatened Species in 2017 [5]. Recent studies on the species have mainly focused on its external morphology [6,7] and the required nutrient levels [8,9] of this species.

The seedlings of *S. cirrhifer* are mostly obtained from wild-catch, as artificial breeding techniques have lacked sufficient success. One possible reason for this challenge in rearing the species is that the early survival rate is relatively low [10], and research related to the early growth, development, and feeding characteristics of the species is limited [11]. Fish species differ in their feeding characteristics, especially with regard to the transformation from endogenous to exogenous nutrition in the larvae. The timing of this transformation is species-specific, as shown in the red sea bream, *Pagrus major* [12], and common snook, *Centropomus undecimalis* [13]. At present, studies on feeding characteristics in the early developmental stages mainly address feeding rhythm, feeding incidence, satiety, and satiety rate [14,15,16].

Starvation—an abnormal physiological feeding state—is a leading cause of mortality in most larvae post hatching. The point-of-no-return (PNR), also termed irreversible starvation, refers to the critical point in time after which young fish can no longer tolerate starvation. When hungry larvae reach the PNR, they are still able to survive for a brief period; however, an estimated 50% of these individuals can no longer resume normal feeding [17]. The PNR is mainly determined by the species, feeding behaviour, and cultivation environment [18,19,20]. Larvae must access exogenous nutrients in a timely manner during the feeding period; otherwise, they are forced to consume their own tissue nutrients to meet the energy demands required for basic metabolism, which then leads to delayed organ development, atrophy, and, ultimately, individual death [21]. A high mortality rate among reared larvae has often been reported during their transition from endogenous to exogenous feeding, also known as the “critical period” [22,23,24]. Previous studies have demonstrated that other critical periods may exist, for example, during incubation, gill filament formation, upstream migration, and metamorphosis [25,26,27]. Therefore, studying the feeding characteristics and rhythms occurring during early development in fish is crucial for the technological progress required to establish successful artificial breeding and seedling production for the improvement of fish stocks.

The objective of this study was to record the growth, development, and feeding characteristics present during early ontogenesis in *S. cirrhifer*. Such data not only fills the theoretical gap regarding the early developmental biology of *S.cirrhifer* but also provides guidelines for general larval rearing and feeding in fish. A better understanding of the interaction between basic biology, feeding needs, and environmental preferences will aid in establishing evidence-based, successful feeding strategies.

## 2. Materials and Methods

### 2.1. Experimental Materials

Wild parental *S. cirrhifer* were obtained from the Yellow Sea (37°16′ N, 121°57′ E). Subsequent, experiments were conducted at the fish-rearing facilities of the Yantai Laizhou Shunchang Aquatic Products Co., Ltd. (Yantai, China), in Shandong Province, China. A sample population of 100 female and 100 male fish were used for artificial propagation. The average body weights of female and male fish were 587.54 ± 86.30 g and 410.23 ± 57.28 g, respectively.

The fish were reared in a recirculating aquaculture system using a cement tank with dimensions of 6 m × 5 m × 1 m, into which an initial stock density of approximately 150 individuals per litre was introduced. During the experimental period, the following ranges of water quality parameters were recorded: a water temperature of 11.2–15.9 °C, pH of 8.0–8.5, dissolved oxygen of 5.5–6.1 mg/L, and salinity of 31.5–32.5 ‰. The fish were fed twice daily with fresh mussel and oyster meat and live sand silkworms to promote gonadal development. After maturation, male and female fish mated naturally. The fertilised eggs were collected and hatched under a constant temperature of 23.6 °C in slow-flowing and aerated sea water. After hatching, the larvae were fed the following items, depending on their growth stage: S-type rotifers at 3–10 days post hatching (dph), L-type rotifers at 8–23 dph, *Artemia* nauplii at 21–38 dph, *Artemia* adults at 35–45 dph, and artificial compound feed after 38 dph. All food items were obtained from Yantai Liuhe Feed Co., Ltd. (Yantai, China).

### 2.2. Measurement of Growth Indices

**Figure 1:**
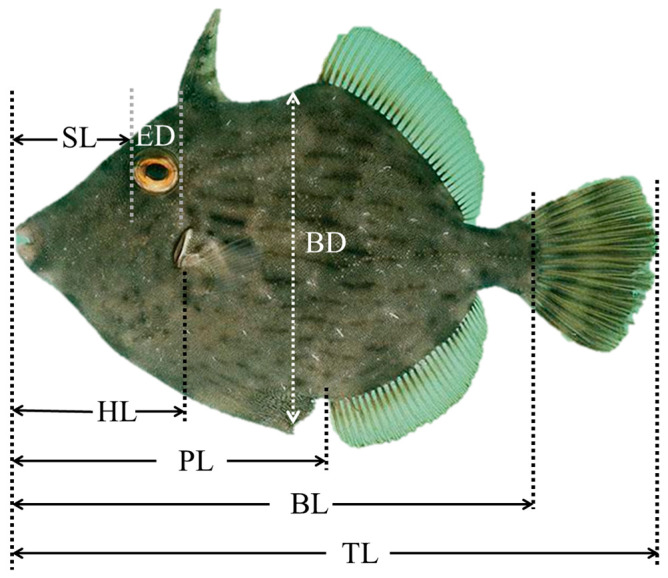
A diagram illustrating the growth indices measured for *Stephanolepis cirrhifer* sample specimens. TL, total length; BL, body length; PL, preanal length; HL, head length; SL, snout length; ED, eye orbit diameter; BD, body depth.

Twenty specimens were retrieved daily at 0–10 dph, every two days at 10–35 dph, and every five days at 35–50 dph. These individuals were anesthetized using MS-222 (Hangzhou Animal Medicine Factory, Hangzhou, China), at the dosages prescribed for various teleosts [28] and a concentration of 100 mg/L. The body weight (BW) of sampled larvae was measured with a precision electronic balance (ME55; Mettler-Toledo International Inc., Zurich, Switzerland). The total length (TL), body length (BL), preanal length (PL), head length (HL), snout length (SL), eye diameter (ED), and body depth (BD) were measured under a dissection microscope (Nikon SMZ800, Nikon Corporation, Tokyo, Japan) (Figure 1).

### 2.3. Yolk Sac and Oil Globule Volume

The major and minor diameters of the yolk sac and the oil globule diameter were measured with a dissection microscope. As per Srithongthum et al. (2020) [29], the yolk sac volume (V_y_, mm^3^) was calculated as:

V_y_ = (4/3)·π·(R/2)·(r/2)^2^,

where R is the major and r the minor diameter of the yolk sac.

Oil globule volume (Vo) was calculated as:

Vo = 4/3·π·(D/2)^3^,

where D is the diameter of the oil globule.

### 2.4. Satiety, Satiety Rate, Feeding Rate, and PNR

Thirty specimens were collected at 3, 4, 5, 8, 12, 25, and 35 dph and euthanized using MS-222 at a concentration of 100 mg/L. The fish digestive tract was excised and dissected to count the food items present. As per Chai et al. (2011) [18], feeding rate was defined as the percentage of feeding individuals among the observed larvae. Satiety was defined as the total number of food items ingested until the larvae either stopped eating or started spitting out food [30]. The satiety rate was defined as the percentage of satiated individuals out of the total number of individuals counted.

The PNR was determined using a series of feeding experiments. Five hundred newly hatched larvae were placed in a 30 L polyethene water tank for starvation cultivation; all other parameters were identical to those used in the experimental pool. Once the larvae were sufficiently developed to open their mouths for food intake, 15 larvae were randomly selected every 12 h and placed in a 1 L beaker; the density of rotifers in the beaker was maintained at 10–15 individuals/mL. After 2 h, the larvae were euthanized using MS-222 at a concentration of 100 mg/L. The fish were dissected, and the feeding rate was measured via the digestive tract content. The PNR was defined as the time point when the feeding rate of the starvation-cultured larvae was less than half of the highest initial feeding rate recorded [17]. 

### 2.5. Diel Feeding Rhythm

The light–dark and feeding cycles are the most important factors that entrain biologi-cal rhythms in animals [31]. A diel feeding rhythm experiment was performed on 9–10 May 2022, as per the design of Fast et al. (1997) [32] and Petit et al. (2003) [33]. At each of 4, 8, 12, 25, and 35 dph, a total of 400 fish were randomly selected from the pool and placed in two 400 L polyethene water tanks (i.e., with 200 fish per tank), marked as A and B. The density of bait organisms in the tanks were maintained at 5–10 individual rotifers/mL and 2–3 *A.* nauplii/mL. Tank A was maintained under 24 h of continuous light, while tank B was exposed to a 12 h light (6:00–18:00) and 12 h dark (18:00–6:00) cycle. Twenty fish were randomly selected from tanks A and B every 3 h, from 6:00 am on the first day to 6:00 am on the second day. The digestive tract of each fry was dissected, and prey organisms therein were classified and counted. Detection was performed continuously in the group exposed to 24 h of light. However, detection was only conducted under light conditions for the group exposed to a 12 h light:12 h dark cycle. The detection was only carried out under the light condition.

### 2.6. Image Processing and Statistical Analysis

All data were analysed using SPSS 26.0 software (IBM, Armonk, NY, USA). Plots were constructed using OriginPro 9.1 (OriginLab, Northampton, MA, USA) and Adobe Photoshop CS6 13.0 software (Adobe Systems, San Jose, CA, USA). The data are expressed as the mean ± standard deviation (SD). The normality of the distribution of continuous data was assessed with Kolmogorov–Smirnov or Shapiro–Wilk tests. Differences between independent samples were assessed using one-way analysis of variance (ANOVA) with Tukey’s honest significant difference (HSD) test. All differences with *p* < 0.05 were considered statistically significant.

## 3. Results

### 3.1. Changes in Growth Indicators from 0 to 50 dph in S. cirrhifer

**Figure 2:**
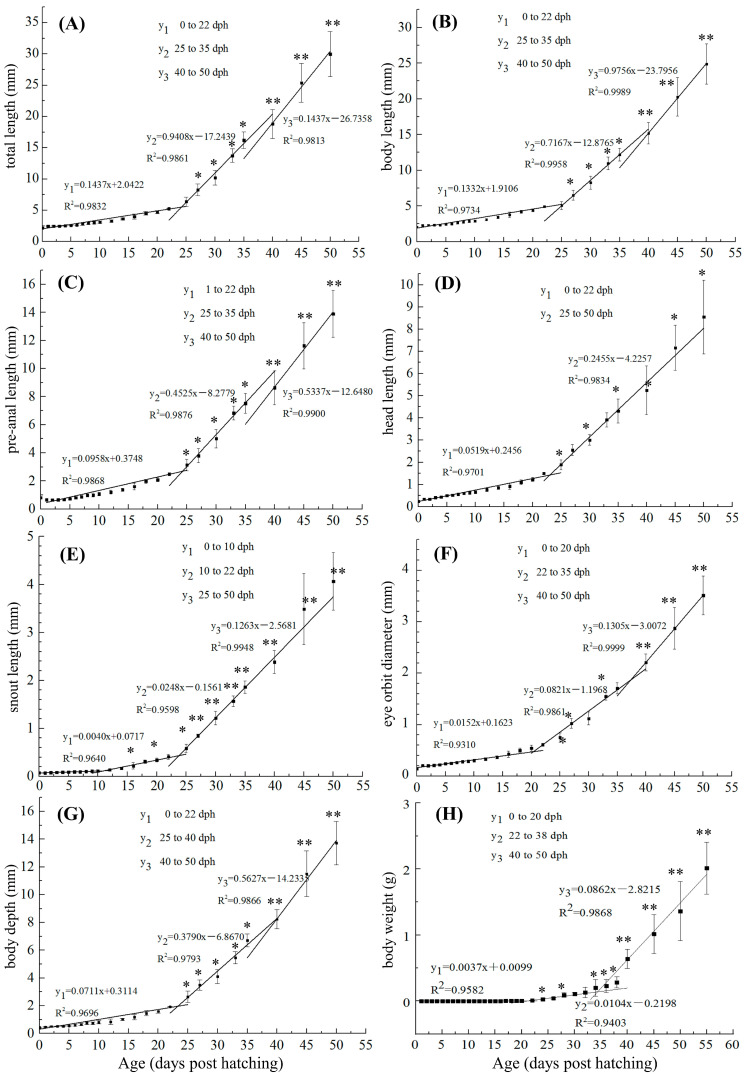
(**A**–**H**) Changes in growth indices from 0 to 50 days post hatching (dph) in *Steph anolepis cirrhifer*. In the formula y = kx + b, x represents the age, y represents the growth indicator, b represents the intercept, and k represents the slope. The asterisks “*” (for y_2_) and “**” (for y_3_) indicate a significant difference from the first growth stage, y_1_ (*p* < 0.05).

The changes in BW, TL, BL, AL, SL, HL, BD, and ED recorded for *S. cirrhifer* are shown in Figure 2. From 0 to 16 dph, no significant differences were observed in any of these growth indices (*p >* 0.05). From 20 dph onwards, all indices began to increase. BW increased significantly (*p* < 0.05) from 2.90 ± 0.62 mg at 20 dph to 41.10 ± 9.89 mg at 26 dph. TL similarly showed a significant (*p* < 0.05) increase from 4.71 ± 0.23 mm at 20 dph to 8.27 ± 0.95 mm at 28 dph. An increase in BL became evident at 30 dph, and increases observed for those of HL, PL, SL, BD, and ED at 28 dph were significant (*p* < 0.05). Furthermore, at 40, 45, and 50 dph, each growth index was significantly higher than its corresponding values at previous time points (*p* < 0.05).

### 3.2. Feeding Rate, Satiety, and Satiety Rate

As shown in Table 1, the feeding rate of the larvae was 73.3% during the initial period of active feeding at 3 dph. Subsequently, the feeding rate gradually increased and reached 100% at 8 dph. The satiety rate was less than 50% at 3–5 dph and clearly increased after 8 dph. In terms of satiety, juveniles required only 5–7 S-rotifers at 3 or 4 dph and 24 L-rotifer at 8 dph. The satiety rate then increased to 32 L-rotifers at 12 dph, 37 *A.* nauplii at 25 dph, and 143 *A.* nauplii at 35 dph.

### 3.3. Absorption of Endogenous Nutrients and PNR

**Figure 3:**
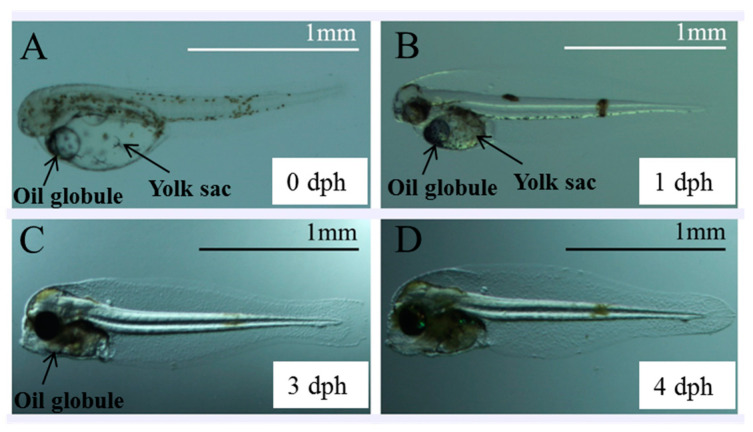
The Yolk sacs and oil globules in larval *Stephanolepis cirrhifer*. (**A**), larval fish at 0 dph; (**B**), larval fish at 1 dph; (**C**), larval fish at 3 dph; (**D**), larval fish at 4 dph.

**Figure 4:**
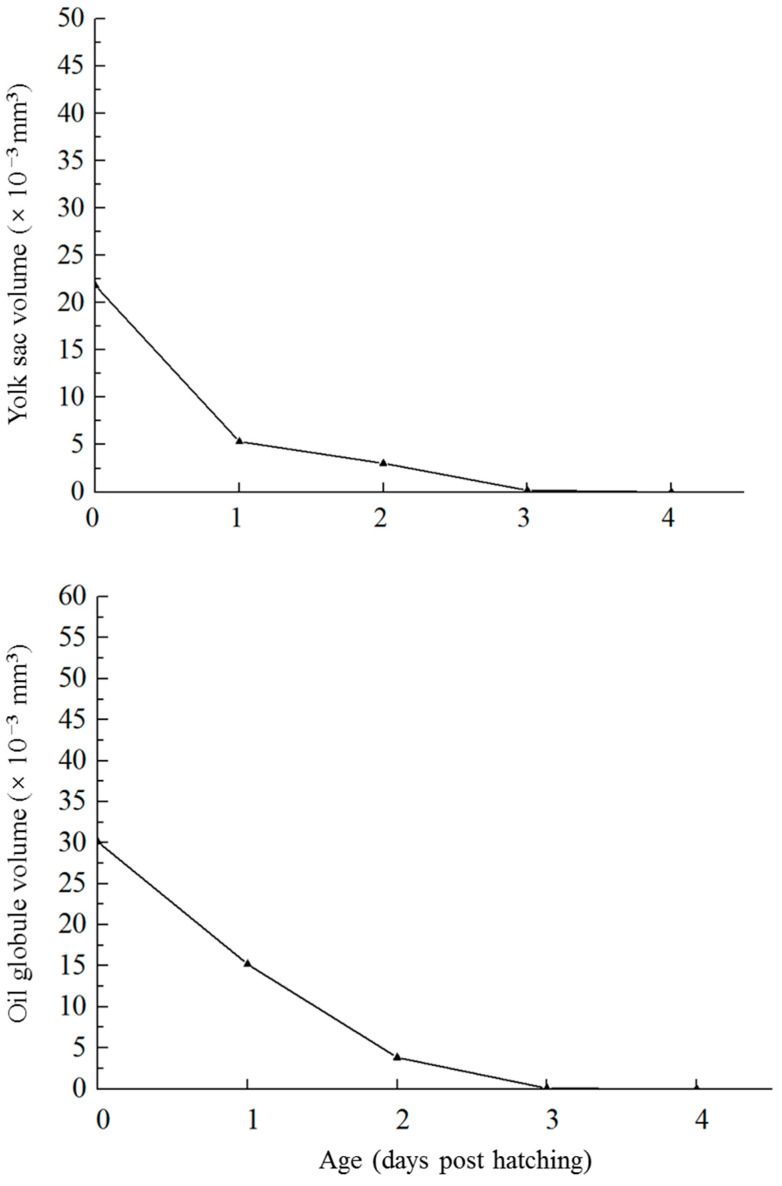
Exhaustion of the yolk sacs and oil globules of larval *Stephanolepis cirrhifer*.

**Figure 5:**
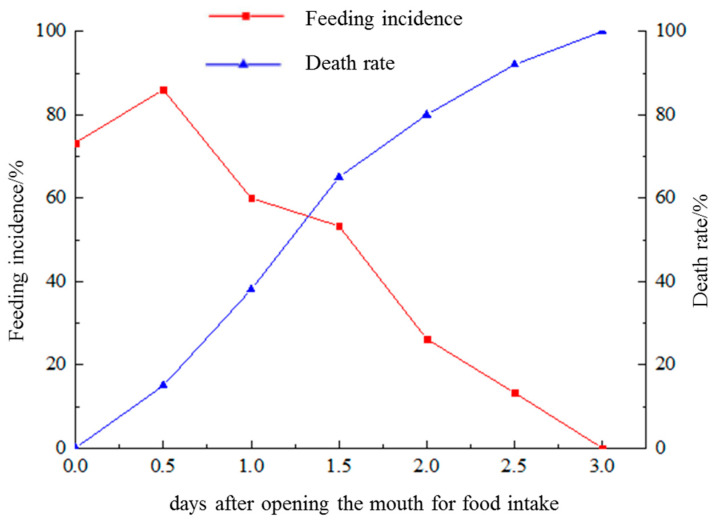
Changes in the feeding (red line) and death rate (blue line) of *Stephanolepis cirrhifer* larvae under starvation.

In newly hatched larvae, the yolk sac volume was 21.81 × 10^−3^ mm^3^, and the oil globule volume was 30.11 × 10^−4^ mm^3^ (Figure 3A). As the larvae developed, the yolk sac and oil globule were absorbed, with more than 99% being absorbed by 3 dph (Figure 3C). After 4 dph, all yolk sacs and oil globules were completely absorbed (Figure 3D). The volume changes of yolk sacs and oil globules were shown in Figure 4. Under starvation conditions, the initial feeding rate of the larvae (with initiation being indicated by the mouth opening) was the highest at 86% on 0.5 dph, and then it decreased to 53.3% by 1.5 dph. After 2 days, the feeding rate decreased further to 26.2%, which was less than half of the previous highest initial feeding rate (Figure 5). These results indicate that the larvae reached the PNR phase at 1.5–2 dph. The feeding rate of starvation-cultured larvae at 2.5 dph was only 13.3%, with a concomitant mortality rate of 80%, and all the fish died after 3 dph.

### 3.4. Diel Feeding Rhythm

**Figure 6:**
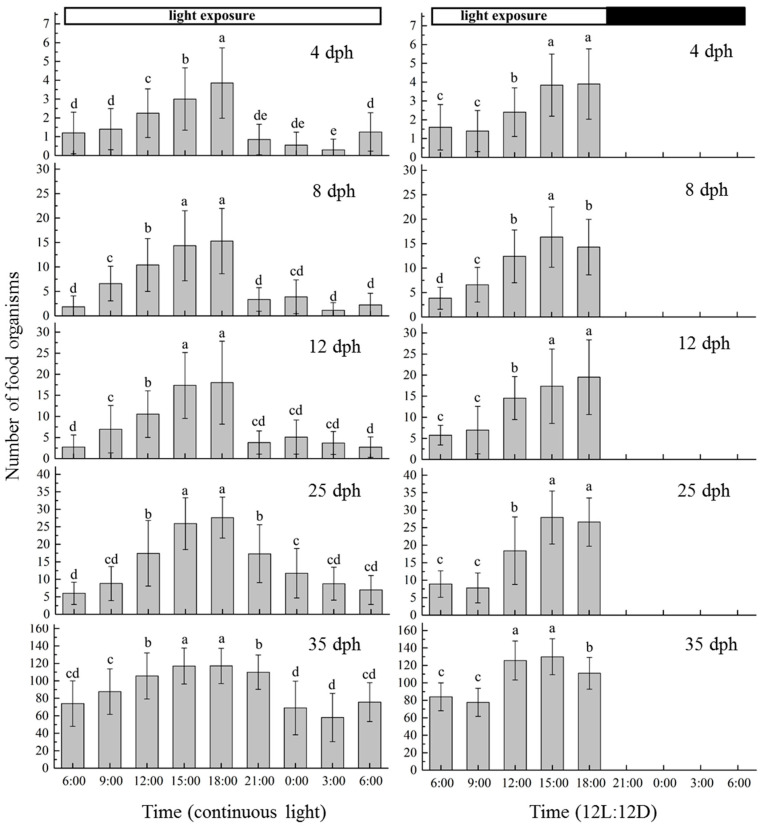
Daily feeding rhythms of *Stephanolepis cirrhifer*. The food organisms at 4 dph, 8 and 12 dph, and 25 and 35 dph are S-Rotifers, L-Rotifers, and *Artemia* salina nauplii, respectively. Different letters above the data bars indicate significant differences (*p* < 0.05).

The changes in the diel feeding rhythm of *Stephanolepis cirrhifer* larvae at 4, 8, 12, 25, and 35 dph are shown in Figure 6. Larvae only fed under light conditions, with the feeding pattern exhibited the distinct feeding peaks and valleys typical of a daytime feeding pattern. Under both of the two different photoperiod conditions, the larvae showed two feeding peaks at 15:00 and 18:00, which showed a significantly higher feeding intensity than all other time points (*p* < 0.05). Under 24 h of continuous light exposure, the larvae exhibited characteristic continuous feeding behaviour; however, this included a feeding trough at 0:00 and 3:00, when the feeding intensity was significantly lower than at other time points (*p* < 0.05).

## 4. Discussion

### 4.1. Growth Indices of the Early Developmental Phase

The growth of juvenile fish is characterised by different stages that manifest during specific turning points during the early stages of growth in teleosts, as previously described for *Limanda yokohamae* [34] and *Chelon labrosus* [35]. In the present study, the growth indices were significantly higher at 20, 24, 28, and 30 dph than at each of the preceding time points. During this period, the fish larvae matured into juveniles, and their prey items concurrently changed from rotifers to *A.* nauplii, which may be related to changes in both morphology and nutrition. A similar phenomenon was observed in the seedling cultivation of *Thamnaconus modestus* [36]. Furthermore, at 40, 45, and 50 dph, each growth indicator was significantly higher than that recorded at each of the preceding time points. During these stages, the juveniles already possessed well-developed digestive organs and were successfully fed with a compound feed, which is more abundant in nutrients than *A.* nauplii and was therefore suitable for rapid growth.

### 4.2. Mixed Feeding Stage, Yolk-Sac Absorption, and PNR during Early Development

The nutritional process during early development is complex in the fish life cycle and consists of endogenous, exogenous, and mixed nutrition [37,38]. The duration of the mixed-nutrition period varies depending on the fish species. For example, in Atlantic salmon, *Salmo salar*, the first feeding of larvae is evident at 41 dph, and the yolk sac is not completely absorbed until 71 dph, with a resulting mixed-nutrition period of up to 30 days [39]. In contrast, in juvenile Japanese anchovy, *Engraulis japonica*, the mouths of the juvenile fish are open and the yolk is already partially absorbed after 1 dph; the yolk is completely absorbed and the mouth fully functional after 2 dph [40]. In other words, the duration of the mixed-nutrition period in this species is only one day. Newly hatched larvae, whose organs are not fully developed, have no active feeding ability, and they rely on the residual yolk sac and oil globules for nutrition, as in the red snapper, *Lutjanus campechanus* [41]. As the yolk sac and oil globule gradually disappear, movement and feeding behaviour develop, along with organ functionality, until the larvae can feed actively and rely on external nutrition (prey items) to provide energy for subsequent development.

In most species, the death rate peaks at the onset of active feeding. After the yolk sac is completely absorbed, juveniles may fail to establish exogenous nutritional orientation and experience progressive hunger [42]. During the transition from endogenous to exogenous nutrition, juveniles have adaptive ecological strategies in response to hunger stress, and they can extend their tolerance to hunger to maintain their development rate [43]. In this state, the larvae begin to consume nutrients derived from their own tissues to meet their energy needs, leading to a decrease in the survival rate of the seedlings during artificial breeding [44]. As a result, if food is supplied too early during seedling cultivation, it is not utilised because the larval mouth crack is not completely formed and the digestive tract is not fully connected; if fed too late, the larvae reach the PNR stage before adequate food is available and begin to lose their feeding ability, resulting in a decreased survival rate. In the present study, *S. cirrhifer*, larvae exhibited a peak in their first feeding under starvation cultivation at 0.5 dph, and thereafter feeding activity began to decline. The PNR stage was recorded from 1.5 to 2 dph. This suggests that the optimal initial feeding time for *S. cirrhifer* is at 1.5–2 dph. The PNR is reported to occur at 15 dph in the Chinese sturgeon, *Acipenser sinensis* [18], and at 50 dph in the silver therapon, *Leiopotherapon plumbeus* [20]. These results indicate that, compared with other fish species, *S. cirrhifer* has a much lower tolerance to starvation.

### 4.3. Feeding Rhythms of Early Development Stage

The feeding activity of fish usually exhibits a specific rhythm [45,46]. This is an active adaptation to their environment during long-term natural evolution. There are four types of daily feeding rhythms: daytime, nighttime, daytime and nighttime, and no obvious rhythm. The goldfish, *Carassius auratus*, mostly feeds during the daytime, but sometimes changes to a nighttime feeding pattern [47]. In the Florida pompano, *Trachinotus carolinus*, feeding activity peaks at dawn and then progressively decreases during the rest of the light period [48]. The nocturnal feeding of zebrafish was found to be particularly peculiar, because feeding was not distributed evenly throughout the night but was concentrated in the last 4 h of the dark phase [49]. These results demonstrate that feeding rhythms vary depending on the fish species and the external environment. In the present study, juveniles of *S. cirrhifer* were fed during the daytime under continuous light conditions. The peak feeding period occurred at 15:00 and 18:00, suggesting a daytime feeding pattern. Therefore, in the breeding and rearing of these larvae, feeding should be implemented during the day, especially between 15:00 and 18:00.

## 5. Conclusions

The growth indices of juvenile *S. cirrhifer* showed significant increases at 20, 24, 28, and 30 dph, suggesting that juveniles possessed well-developed digestive organs in this time period and successfully fed on compound feed, suitable for rapid growth. There may be a short mixed-nutrition period (both endogenous and exogenous) at 3–4 dph. The PNR stage is likely to occur from 1.5 to 2 dph. Furthermore, *S. cirrhifer* should be fed during the day, ideally between 15:00 and 18:00. 

As the global numbers and quality of *S. cirrhifer* are gradually decreasing, it is essential to establish breeding protocols for this species in which exogenous nutrients are provided as soon as is feasible and to conduct initial feeding in a timely manner, thereby maintaining normal growth and development. The results of this study provide guidance for such optimised larval rearing and feeding to improve seedling survival rates in *S. cirrhifer*. 

## Figures and Tables

**Table 1:** Feeding rate, satiety, and satiety rate of larval and juvenile *Stephanolepis cirrhifer*.

dph	Sampling Numbers	Feeding Rate (%)	Satiety Prey Item	Satiety Rate (%)
3	30	73.3	5 (S-Rotifer)	33.33
4	30	90	7 (S-Rotifer)	36.67
5	30	96.7	13 (S-Rotifer)	43.33
8	30	100	24 (L-Rotifer)	76.67
12	30	100	32 (L-Rotifer)	83.33
25	30	100	37 (*Artemia salina* nauplii)	83.33
35	30	100	143 (*A. salina* nauplii)	100

## Data Availability

The data that support the findings of this study are available from the corresponding author upon reasonable request.
